# Influence of Morphine on Pericyte-Endothelial Interaction: Implications for Antiangiogenic Therapy

**DOI:** 10.1155/2012/458385

**Published:** 2012-01-17

**Authors:** Kathryn Luk, Sonja Boatman, Katherine N. Johnson, Olivia A. Dudek, Natalie Ristau, Derek Vang, Julia Nguyen, Kalpna Gupta

**Affiliations:** Vascular Biology Center and Division of Hematology, Oncology and Transplantation, Department of Medicine, University of Minnesota Medical School, Mayo Mail Code 480, 420 Delaware Street SE, Minneapolis, MN 55455, USA

## Abstract

Morphine stimulates tumor angiogenesis and cancer progression in mice. We examined if morphine influences endothelial-pericyte interaction via platelet-derived growth factor-BB (PDGF-BB) and PDGF receptor-*β* (PDGFR-*β*). Clinically relevant doses of morphine stimulated PDGF-BB secretion from human umbilical vein endothelial cells and activated PDGFR-*β* and mitogen-activated protein kinase/extracellular signal-regulated kinase (MAPK/ERK) phosphorylation in human pericytes. These *in vitro* effects of morphine were translated into promotion of tumor angiogenesis in a transgenic mice model of breast cancer when treated with clinically used dose of morphine. Increased vessel-associated immunoreactivity of desmin and PDGFR-*β* was observed on pericytes in tumors of morphine-treated mice. These data suggest that morphine potentiates endothelial-pericyte interaction via PDGF-BB/PDGFR-*β* signaling and promotes tumor angiogenesis, pericyte recruitment, and coverage of tumor vessels. We speculate that morphine may impair the effectiveness of antiangiogenic therapy by influencing vascular pericyte coverage.

## 1. Introduction

Angiogenesis, sprouting of new blood vessels from the existing vessels, is critical for cancer progression and metastases [1]. Endothelial cells, the building blocks of blood vessels, and endothelial cell-specific cytokine vascular endothelial growth factor (VEGF) and its receptors have therefore been a target of anticancer therapies [2–4]. Several VEGF and endothelial cell specific therapies are either in clinical use or clinical trials. However, drug resistance and ineffectiveness are a major challenge limiting the success of these promising new drugs. Recent studies suggest that vasculature is not merely an endothelial structure, rather it is closely associated with mural cells including pericytes and vascular smooth muscle cells (VSMCs) [5–7]. The exact role of pericyte coverage of tumor vasculature is not clear, but paradoxical roles are proposed that favor inhibition of angiogenesis on one hand and a barrier to antiangiogenic therapy on the other [4, 8, 9].

Pericyte coverage of the vascular endothelium is controlled by several cytokines including the platelet-derived growth factors (PDGFs) and VEGF [10, 11]. PDGF-BB secreted by endothelial cells acts as an attractant to recruit PDGFR-*β*-expressing pericytes and pericyte progenitor cells to the endothelium [12]. In turn, pericytes provide guidance to endothelial sprouts, scaffolding for the vasculature to grow, and stabilize the vessel wall [5, 13, 14]. Thus, endogenous and exogenous molecules that influence pericyte-endothelial interaction may influence tumor angiogenesis and interfere with therapies directed towards them. For example, morphine stimulates the expression of PDGF-BB in human brain microvascular endothelial cells (HBMECs) [15] and coactivates PDGFR-*β* signaling in the mouse retinal microvascular endothelial (mREC) and mesangial cells (specialized mural cells) in the kidney [16, 17].

Morphine used in clinically relevant doses promotes angiogenesis *in vitro* and *in vivo* and increases vascular permeability [18–20]. This proangiogenic activity of morphine is translated into promotion of breast and lung cancer in mice [18, 19, 21]. Additionally, morphine promotes breast and lung cancer cell proliferation and migration. Opioid receptors (ORs) particularly mu opioid receptor (MOR) mediate the analgesic effect of morphine and are highly expressed in human lung cancer [21–23]. Morphine and its congeners are used to treat pain due to cancer, particularly in the advanced stages of malignancy when most of the therapies are ineffective. It is likely that morphine influences endothelial-pericyte interaction and may further contribute to ineffectiveness of targeted therapies.

Therefore, we examined morphine-induced endothelial and pericyte-specific activity mediated by PDGF-BB/PDGFR-*β* signaling. We used primary human umbilical vein endothelial cells (HUVECs) and human placenta-derived pericytes for *in vitro* studies and a transgenic mouse model of breast cancer, which mimics the evolutionary spectrum of human disease. We found that morphine stimulates PDGF-BB secretion by HUVEC and phosphorylation of PDGFR-*β*, mitogen-activated protein kinase/extracellular signal-regulated kinase (MAPK/ERK), and signal transducer and activator of transcription 3 (STAT3) in pericytes. Complementary to these *in vitro* observations, morphine in clinically used doses increased desmin- and PDGFR*β*-positive cells in the tumor vasculature of mice, suggestive of increased proliferation and/or recruitment of vessel-associated pericytes.

## 2. Materials and Methods

### 2.1. Tumor Model and Drug Treatments

Female transgenic mice carrying a rat C3(1) simian virus 40 large tumor antigen (C3TAG) fusion gene that develop highly invasive breast tumors were used. Female C3TAG mice show the evolutionary spectrum of human infiltrating ductal carcinoma [24]. These mice develop ductal epithelial atypia at 8 weeks, progression to intraepithelial neoplasia at 12 weeks (resembling human ductal carcinoma in situ), and invasive carcinoma and grossly palpable tumors at 16 weeks. Tumors predominantly metastasize hematogenously to the lungs, and also to the liver, adrenals, and heart. By 6 months of age, all the mice die because of universal development of multifocal mammary adenocarcinomas. We have used this model in previous studies to target tumor angiogenesis using blood outgrowth endothelial cells expressing sFlt1 [25]. Three-month-old C3TAG mice were subcutaneously injected with morphine sulfate (Baxter Esilerderle Healthcare, Cherry Hill, NJ) at 0.5 mg/Kg/d for 2 wks and the dose escalated every two weeks to 0.75 mg/Kg/d, 1.0 mg/Kg/d, 1.25 mg/Kg/d, and 1.5 mg/Kg/d, or with PBS, for a period of seven weeks. All reagents were from Sigma-Aldrich, St. Louis, MO, unless specified.

### 2.2. HUVEC Culture

Human umbilical vascular endothelial cells (HUVECs) were isolated from umbilical cords and cultured as previously described [26]. Complete HUVEC culture medium (CHCM) consisted of medium 199 (Life Technologies, Inc., Gaithersburg, MD), 40 *μ*g/mL heparin sodium salt, 15% fetal bovine serum, 2.4% 200 mM L-glutamine, 100 units/mL penicillin, 100 units/mL streptomycin, 100 units/mL fungizone, 0.04% freshly thawed ENDO-GRO, and 0.1% sodium pyruvate. Primary HUVECs between passages one and three were used for all experiments. HUVECs were cultured in serum and growth factor-free medium (SFM) to examine the effect of morphine on PDGF-BB expression and for signaling studies. SFM consisted of medium MCDB 131 (Life Technologies, Inc., Gaithersburg, MD), dibutyryl cAMP, heparin, L-glutamine, Pen/Strep/Fungizone, and hydrocortisone as described [18].

### 2.3. Pericyte Culture

Human pericytes from placenta were purchased from PromoCell (PromoCell, Heidelberg, Germany) and cultivated per the manufacturer's instructions in pericyte growth medium. Pericytes were serum and growth factor starved in pericyte serum and growth factor-free medium (PSFM). PSFM consisted of medium 199 (Life Technologies, Inc., Gaithersburg, MD), 0.5% fetal bovine serum, 100 units/mL penicillin, 100 units/mL streptomycin, 100 units/mL fungizone, and 2 mM L-Glutamine.

### 2.4. ELISA

HUVEC were serum and growth factor starved in SFM overnight followed by incubation with different concentrations of morphine indicated in the figures for an additional 48 hrs. The supernatant from HUVEC was analyzed for PDGF-BB using an ELISA Kit (RayBiotech, Norcross, GA). Absorbance was read at 450 nm using an ELISA reader (Synergy HT, Winooski, VT). The concentration of PDGF-BB in the supernatant was calculated using the standard curve prepared in parallel with each experiment. SFM cultured without HUVEC in parallel to the experiment was used as a blank/negative control.

### 2.5. Western Blot Analysis

Pericytes were serum starved overnight as described and stimulated with 0.1 *μ*M morphine or 20 ng/mL of PDGF-BB. Cell lysates were prepared as described by us earlier using a cocktail of protease inhibitors [18]. Protein lysates containing 100 *μ*g of protein were separated on a 3–15% gradient SDS-PAGE gel and then transferred to a polyvinylidene fluoride membrane (Immobilon; Millipore, Bedford, MA). Protein bands were detected using 1 : 250 phospho-PDGFR-*β* (Upstate, Lake Placid, NY), 1 : 500 PDGFR-*β*, 1 : 1000 phospho-STAT3, 1 : 1000 STAT3, 1 : 1000 phospho-MAPK/ERK, and 1 : 1000 MAPK/ERK (all from Cell Signaling Technology, Danvers, MA). Alkaline phosphate-conjugated secondary antibodies (1 : 5000, Santa Cruz Biotechnology, Santa Cruz, CA) and ECF system (Amersham Bioscience, Buckinghamshire, UK) were used to detect chemiluminescent signals on a Storm 860 Phosphorimager (Molecular Dynamics, Sunnyvale, CA). Protein bands were quantitated by densitometric analysis using ImageJ Software (National Institutes of Health, Bethesda, MD).

### 2.6. Immunofluorescent Staining

Tumors were frozen in liquid nitrogen, embedded in optimal cutting temperature compound (OCT), and cut into 6 *μ*M cryosections. Sections were fixed in 4% paraformaldehyde and immunostained using the following primary antibodies at the indicated dilutions: 1 : 100 rabbit anti-PDGFR-*β* (Upstate, Lake Placid, NY); 1 : 100 mouse antismooth muscle actin (*α*-SMA; Sigma, St. Louis, MO); 1 : 50 goat antidesmin (Santa Cruz); 1 : 50 rat anti-CD31-FITC (BD Pharmingen, San Diego, CA). Species-specific secondary antibodies conjugated with Cy3 or TRITC were used at the following dilutions: 1 : 400 donkey anti-rabbit IgG-Cy3 (Jackson Laboratories, West Grove, PA) and 1 : 50 donkey anti-goat IgG-TRITC (Santa Cruz). In addition, isotype-matched IgG was used as control (Santa Cruz Biotechnology, Santa Cruz, CA). Fluorescent images were visualized and obtained using an Olympus IX70 epifluorescent microscope with an attached Olympus DP70 digital camera (Olympus America Inc., Center Valley, PA).

### 2.7. Quantitation of Immunoreactive Pixels and Tumor Angiogenesis

Pericyte markers, desmin, *α*-SMA, and PDGFR-*β*, were examined in relation to endothelial cell marker CD31. Superimposed images were analyzed with Adobe Photoshop to calculate the ratio of desmin, *α*-SMA, or PDGFR-*β* relative to CD31-positive cells. Ratios were based on the fluorescent intensity of the proteins. For morphometric analysis of tumor angiogenesis, CD31-positive images were binarized and skeletonized using Adobe Photoshop and the Image Processing Tool-Kit Plug-in Functions for Adobe Photoshop (Reindeer Games, Asheville, NC), and total lengths, ends, and nodes of vessels were quantified as described by us earlier [18].

### 2.8. Statistical Analysis

All data are expressed as mean ± SEM. All statistical analyses were performed using Prism software (GraphPad Prism Inc., San Diego, CA). Significance was determined using unpaired, Student's *t*-tests. *P* < 0.05 was considered significant.

## 3. Results

### 3.1. Morphine Stimulates PDGF-BB Secretion by HUVEC

**Figure 1:**
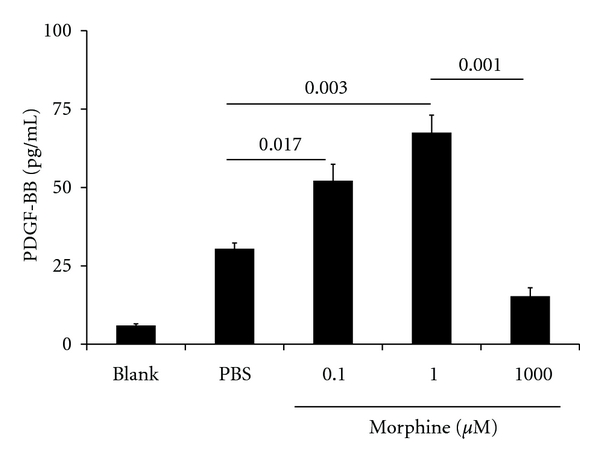
Morphine stimulates PDGF-BB release from endothelial cells. PDGF-BB was analyzed in the cell culture supernatants of HUVEC incubated with different concentrations of morphine or PBS in serum- and growth factor-free medium for 48 h at 37°C. In parallel, serum- and growth factor-free medium was incubated in flasks without cells to serve as blank. A dose-dependent increase in PDGF-BB is seen between 0.1 and 1 *μ*M morphine, whereas no statistically significant increase occurred with 1 mM morphine, as compared to PBS. Each bar is the mean ± SEM of three separate experiments from 3 different cultures of HUVEC.

Morphine induces PDGF-BB expression in HBMECs [15], but this needs to be secreted to have a paracrine effect on pericytes. We examined if PDGF-B was secreted into the culture supernatants by HUVEC stimulated with morphine for 48 h. Morphine at the doses of 0.1 and 1 *μ*M stimulated about a 2-fold increase in the secretion of PDGF-BB in the culture medium as compared to PBS (Figure 1). However, 1 mM concentration of morphine did not have a significant effect on PDGF-BB secretion as compared to PBS. Phase contrast microscopy and Trypan blue staining of HUVEC incubated with 0.1 and 1 *μ*M morphine showed that more than 99% cells were alive and appeared normal (data not shown). In contrast, more than 99% of HUVECs were dead after incubation with 1 mM morphine. Morphine concentrations at 0.1 and 1 *μ*M are consistent with the observed plasma/serum concentration of diverse patient population treated with morphine, which ranges between 2 nM and 3.5 mM [27, 28]. Earlier studies from our laboratory demonstrated that 1 mM morphine was cytotoxic to human dermal microvascular endothelial cells (HDMECs) [18]. Thus, clinically relevant concentration of morphine stimulates PDGF-BB secretion from endothelial cells, a key step in endothelial-pericyte interaction.

### 3.2. Morphine Activates PDGFR-*β*, MAPK/ERK, and Stat3 Signaling in Pericytes

**Figure 2:**
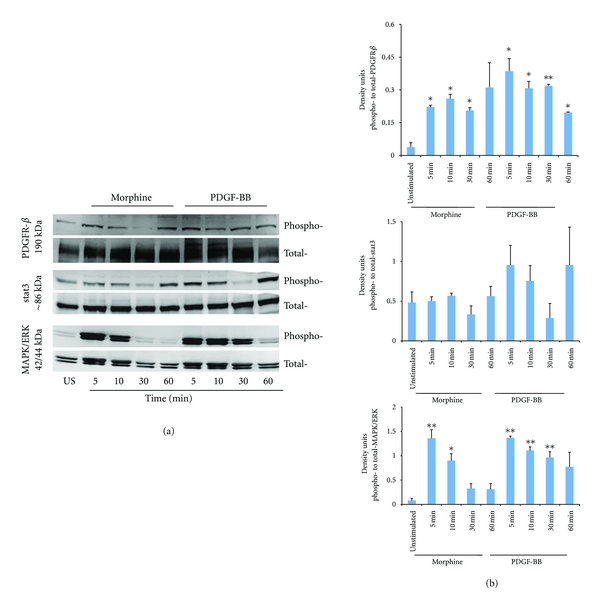
Morphine coactivates PDGFR-*β* phosphorylation and MAPK/ERK signaling in human pericytes. Human placenta-derived pericytes were incubated with 0.1 *μ*M morphine or 20 ng/mL PDGF-BB for indicated time or with PBS, followed by cell lysis using protease inhibitors. Lysates containing 100 *μ*g protein were resolved on 3–15% SDS gel and Western blotted as described in the methods. (a) Representative image of five different Western blots for phospho- and total-PDGFR-*β*, MAPK/ERK, and STAT3 is shown. (b) Densitometric analysis of protein bands is represented as a ratio of phospho- to total-protein for each protein. Each bar is the mean ± SEM of five different blots. *P < 0.01, **P < 0.001, for each time point compared to unstimulated in each graph.

Proliferation, recruitment, and endothelial interaction of pericytes with endothelium are dependent upon PDGFR-*β* signaling. We observed that 0.1 *μ*M morphine as well as 20 ng/mL PDGF-BB stimulates sustained activation of PDGFR-*β* phosphorylation on pericytes from 5 min to 60 min of incubation (Figures 2(a) and 2(b)). Interestingly, both morphine and PDGF-BB significantly stimulated MAPK/ERK phosphorylation in a time-dependent manner, which returned to baseline after 60 min of incubation. On the other hand, both morphine and PDGF-BB stimulated the phosphorylation of STAT3, but it was not statistically significant. Therefore, both morphine and PDGF-BB stimulate PDGFR-*β* and MAPK/ERK signaling in pericytes.

### 3.3. Morphine Promotes Angiogenesis in Tumors of C3TAG Mice

**Figure 3:**
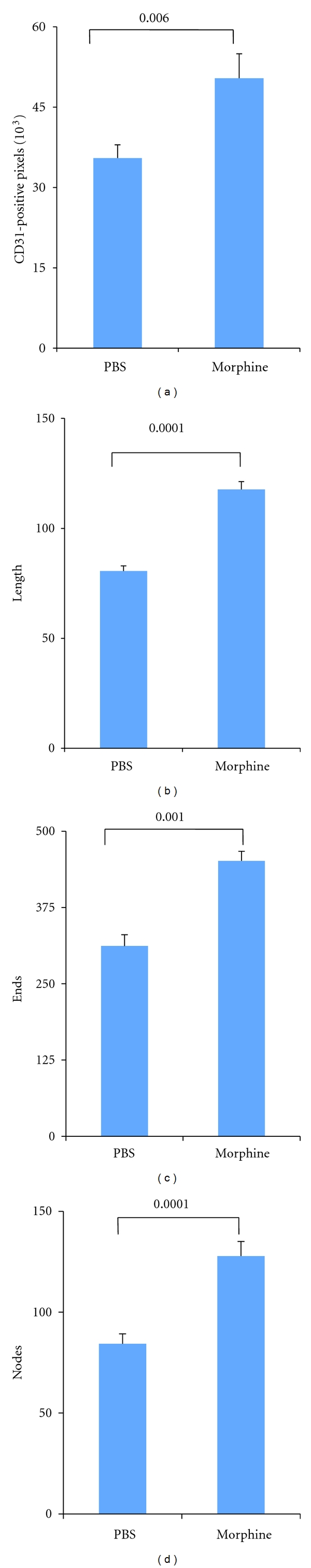
Morphine stimulates tumor angiogenesis. C3TAG mice at 3 months of age were subcutaneously injected with morphine at 0.5 mg/Kg/d for 2 wks, and the dose was escalated every two weeks to 0.75 mg/Kg/d, 1.0 mg/Kg/d, 1.25 mg/Kg/d, and 1.5 mg/Kg/d, or with PBS, for a period of seven weeks. Sections of tumors larger than 1 cm × 0.5 cm were immunostained with anti-CD31-FITC, followed by morphometric analysis to quantify different parameters specific to tumor angiogenesis. (a) CD31-positive pixels indicate total pixels for CD31 immunostaining per image. (b) Length suggests the total length of vessels per image. (c) Ends denote the number of vessels per image. (d) Nodes suggest the number of branch points in an image. Each bar represents mean ± SEM of sections from five different tumors obtained from 5 different mice per treatment.

Three-month-old C3TAG mice bearing multiple breast tumors, which grow spontaneously, were treated with clinically relevant escalating dose of morphine, for seven weeks. Multiple tumors in different sizes were dissected out, but only tumors about 1 cm × 0.5 cm were analyzed for tumor angiogenesis (Figures 3(a)–3(d)). Morphometric analysis was performed on tumor sections stained with anti-CD31-FITC. Different parameters of angiogenesis analyzed included vessel density (a), total length of vessels (b), number of vessels (ends, (c)), and branching (nodes, (d)). A significant increase was observed in each of these parameters in morphine-treated mice as compared to PBS-treated mice. Excessive branching (nodes) and increased number of vessels is a typical feature of disorganized growth of vasculature in tumors. Morphine, therefore, further augments tumor angiogenesis.

### 3.4. Morphine Treatment Results in Increased Desmin Immunoreactivity but Does Not Influence *α*-SMA Immunoreactivity in Tumors

**Figure 4:**
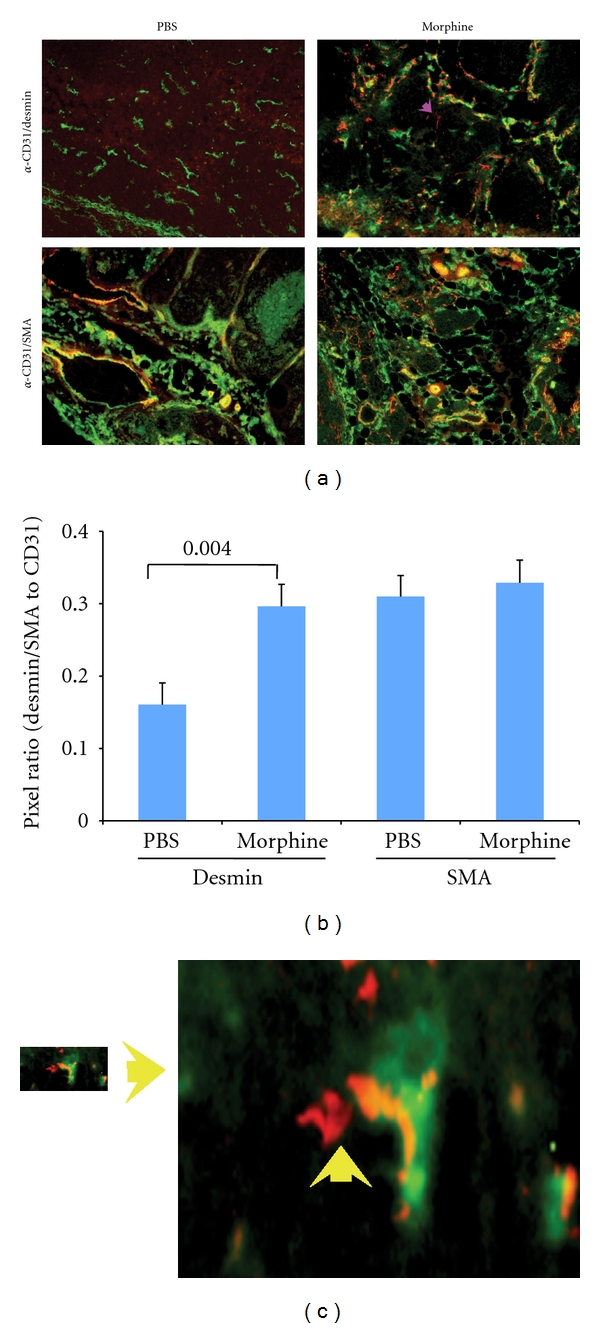
Morphine stimulates vessel-associated Desmin expression in mouse tumors. C3TAG mice were treated with morphine or PBS as described in Figure 3 and the methods. (a) Upper row shows tumor sections stained with anti-CD31 for vasculature (green) and desmin (red). Morphine-treated tumors show strong desmin (red) staining associated with tumor endothelium (green) as well as independent of vasculature (magenta arrow). Orange staining suggests an overlap between red and green staining for desmin and vasculature, respectively. Lower row shows strong costaining of *α*-smooth muscle actin (SMA, red) with CD31 (green), in both PBS and morphine-treated mice tumors. Magnification ×150. Each image represents 5 different tumors from 5 different mice per treatment. (b) Ratios of desmin to CD31- and *α*-SMA to CD31-immunoreactive pixels are shown. A significant difference is observed in desmin/CD31 ratio between morphine and PBS treatment but not in *α*-SMA/CD31 ratio. Each bar represents mean ± SEM of immunoreactive pixels from five tumors (3 different sections of each tumor) obtained from 5 different mice per treatment. (c) Enlargement of area shown with yellow arrow in (a) for CD31/desmin staining in morphine-treated mice. It shows colocalization of desmin staining in the sprouting endothelial cell and in the tip cells.

Tumors from C3TAG mice treated with morphine as described above were costained with anti-CD31-FITC (green vasculature) and desmin (red) or with anti-CD31-FITC (green) and *α*-SMA (red). Immunofluorescent images show a significant increase in desmin immunoreactivity in morphine treated as compared to PBS-treated mouse tumors (Figure 4(a), top row). Most of the desmin staining colocalized with CD31-postive endothelium in a random fashion and showed a significant increase with morphine treatment as compared to PBS (Figure 4(b)). In some areas, desmin staining is independent of CD31 staining (magenta arrow in (a)). Some strongly desmin-positive (red) cells also appeared on the vessel sprouts and tip cells (yellow arrow and enlarged region shown separately in Figure 4(c)) indicative of supporting the formation and guidance of new vessels. In contrast, *α*-SMA immunoreactivity was strong in both morphine- and PBS-treated tumors without any significant difference between the two treatment groups (Figures 4(a) and 4(b)). Notably, all a-SMA immunoreactivity colocalized with vascular endothelium.

### 3.5. Increased Expression of Vascular PDGFR-*β* Immunoreactivity in Tumors of Mice Treated with Morphine

**Figure 5:**
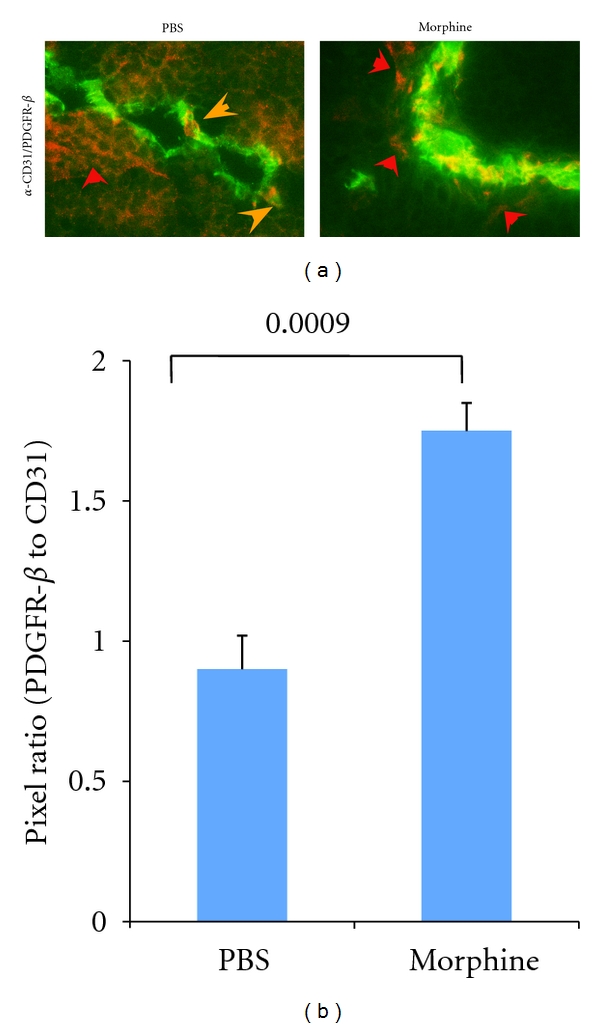
Morphine stimulates vessel-associated PDGFR-*β* expression in mouse tumors. C3TAG mice were treated with morphine or PBS as described in Figure 3 and the methods. (a) Tumor sections were immunostained with anti-CD31 (green) and anti-PDGFR-*β* (red). Vasculature from morphine-treated mouse tumors shows strong costaining for PDGFR-*β*, whereas PDGFR-*β* staining is predominantly observed in nonvascular compartments of PBS-treated mouse tumors. Vessel-associated PDGFR-*β* is observed only near the branch points of vasculature in PBS-treated group (orange arrows). Each image represents 5 different tumors from 5 different mice per treatment. Magnification ×900. (b) Ratios of PDGFR-*β* to CD31, immunoreactive pixels are shown. A significant difference is observed in PDGFR-*β*/CD31 ratio between morphine and PBS treatment. Each bar represents mean ± SEM of immunoreactive pixels from five tumors (3 different sections of each tumor) obtained from 5 different mice per treatment.

Tumor sections of mice treated with morphine show strong costaining of PDGFR-*β* in association with endothelium, which appears orange due to the overlaying of red and green images of PDGFR-*β* and CD31, respectively (Figure 5(a)). Quantitatively also vessel-associated PDGFR-*β* immunoreactivity was significantly higher in morphine as compared to PBS-treated mouse tumors (Figure 5(b)). Most of the PDGFR-*β* immunoreactivity colocalized uniformly with vessels and in the area surrounding the vasculature (red arrows) in morphine treated mice. In contrast, in PBS-treated mice, most of the PDGFR-*β* immunoreactivity colocalized with nonvascular cells, likely with tumor cells. Of note, specific colocalization of PDGFR-*β* was seen on vascular sprouts (orange arrows) and at vessel branch points, in PBS group. Thus, it appears that in the tumors of these mice, PDGFR-*β* is associated with vessel branching and sprouts, whereas morphine treatment increases endothelium-associated pericyte density and pericyte coverage of vasculature.

## 4. Discussion

Clinically used doses of morphine/opioids act on endothelium and tumor cells resulting in tumor progression *in vitro* and *in vivo* experimental studies [18, 21, 22, 29–31]. Significantly higher expression of MOR on human lung cancer tissue as compared to nonmalignant tissue in the same organ complements this activity of morphine [21–23]. Therefore, this study was undertaken to examine if pericytes were directly or indirectly influenced by clinically relevant doses of morphine. We observed that morphine stimulated PDGF-BB secretion by endothelial cells, a critical mediator of endothelial-pericyte crosstalk, thus indirectly influencing pericyte activity. Morphine also activated PDGFR-*β* signaling and MAPK/ERK phosphorylation on human pericytes. These activities of morphine on endothelial cells and pericytes correlate with an increase in angiogenesis, vessel associated-desmin and -PDDGFR-*β* expressing pericytes in transgenic mice with breast cancer. Our observations on morphine-induced vascular-pericyte interaction may have implications upon the effectiveness of antiangiogenic therapy.

PDGF-BB plays a central role in the recruitment and growth of pericytes and in endothelial-pericyte interaction in a paracrine manner [5, 13]. In this relationship, endothelial cells secrete PDGF-BB, which acts upon the pericytes and progenitor cells and recruits them to the endothelium. Increased secretion of PDGF-BB by HUVEC when stimulated with 0.1 and 1 *μ*M morphine demonstrates that morphine plays a salutary role in HUVEC-pericyte interaction. However, 1 mM morphine did not have an effect on PDGF-BB secretion by HUVEC. It is noteworthy that in patients treated with a range of morphine doses for diverse conditions including cancer, morphine concentration ranged between 2 nM and 3.5 *μ*M in the plasma/serum [27, 28], which are in the range of 0.1 and 1 *μ*M concentration showing stimulation of PDGF-BB secretion by HUVEC. The 1 mM concentration of morphine is highly unlikely to be present in the plasma of patients, because an extremely high dose of morphine will be required to achieve this plasma concentration, which in turn may have severe side effects and therefore not used clinically. We demonstrated earlier that 1 mM morphine was cytotoxic to HDMEC [18], and in the present study, we found that HUVECs incubated with 1 mM morphine for 48 h were not alive. Our observation of PDGF-BB secretion by HUVEC in this study is further supported by an increase in expression of PDGF-BB in HBMEC by 10^−7 ^M morphine, but not by 10^−5 ^M morphine [15]. Together, these data suggest that clinically relevant dose of morphine has a stimulatory effect on PDGF-BB production by endothelial cells, which can act in an autocrine and paracrine manner via PDGFR-*β* to promote angiogenesis and pericyte growth and recruitment.

PDGF-BB is known to activate PDGFR-*β* and several downstream signaling pathways that promote cell proliferation, survival, and differentiation, including MAPK/ERK and STAT3 [14, 32, 33]. We found that morphine coactivates VEGFR2 and PDGFR-*β* in mouse retinal microvascular endothelial cells (mRECs) which immunoprecipitated with MOR [16]. Our earlier studies also showed morphine-induced MAPK/ERK, Stat3, and Akt phosphorylation in mREC and HDMEC [16, 18]. In HBMECs, also morphine activated MAPK/ERK and PKB/Akt phosphorylation [15]. More recently, we observed that morphine coactivates PDGFR-*β* signaling in kidney mesangial cells *in vitro* and *in vivo* [17]. MOR silencing on kidney mesangial cells led to a significant decrease in morphine-induced phosphorylation of PDGFR-*β*, MAPK/ERK, Stat3, and PKB/Akt, suggestive of MOR-PDGFR-*β* crosstalk. This is highly significant considering that MOR agonist drugs including morphine are used to treat pain in cancer and that PDGFR-*β* signaling is involved in pericyte growth and recruitment. Our observations herein that morphine activates PDGFR-*β* and MAPK/ERK phosphorylation in pericytes to the same extent as that induced by PDGF-BB suggest that morphine may increase pericyte recruitment to the endothelium and also increase tumor angiogenesis.

Consistent with morphine-induced secretion of PDGF-BB by HUVEC and activation of PDGFR-*β* and MAPK/ERK signaling in pericytes, we observed increased angiogenesis in the tumors of C3TAG mice treated with morphine using clinically relevant doses. Morphine-induced tumor angiogenesis is in agreement with morphine-induced angiogenesis *in vitro* and *in vivo* and promotion of breast and lung tumors in mice [18–22]. Morphine-induced angiogenesis was replete with excessive vessel branching and a significantly larger number of vessels, typical of tumor angiogenesis. These data demonstrate that morphine promotes angiogenesis in a breast cancer model, which recapitulates the evolutionary spectrum of human breast cancer. Together, the promotion of angiogenesis and PDGF-BB/PDGFR-*β* induced endothelial-pericyte interaction promoted by morphine may influence antiangiogenic therapy.

 Increased tumor angiogenesis in morphine-treated mice was accompanied by increased vessel-associated desmin-expressing pericytes, but not *α*-SMA-expressing pericytes. It is believed that *α*-SMA is not expressed on pericytes associated with normal capillaries, which express desmin, while vSMCs on arterioles and pericytes on venules express desmin as well as *α*-SMA [34, 35]. On the other hand, *α*-SMA is suggested to be a marker of pericytes [36]. Irrespective of the treatment, all tumor sections showed a strong expression of *α*-SMA on tumor vessels. It is therefore likely that *α*-SMA is strongly expressed on certain type of vessels in this tumor model, which appear to be similar to arterioles and venules and are not influenced by morphine. Notably, strong desmin immunoreactivity in tumors of morphine-treated mice colocalized with endothelial sprouts and tip cells and in close proximity to endothelial cells. This is an indication of increased pericyte differentiation and recruitment induced by morphine in the vicinity of endothelium. Similarly, cells expressing PDGFR-*β* increasingly colocalized with vasculature in morphine group and in close vicinity to endothelium, further demonstrating increased pericyte recruitment and vascular coverage. Indeed increased PDGFR-b signaling leads to increased pericyte coverage of the vasculature [14]. Interestingly, in PBS-treated mice, vessel-associated PDGFR-*β*-expressing pericytes were few, and were sparsely located at vessel branch points and on the vascular sprouts. Appreciably high PDGFR-*β* expression was observed on nonendothelial cells, perhaps on tumor cells, in PBS-treated mice. Increased microvessel density and thicker PDGFR-*β*-expressing pericyte coverage were associated with highly metastatic human KM12SM colon cancer cecal tumors in nude mice as compared to low metastatic KM12C cell tumors [37]. Therefore, increased vessel-associated pericyte coverage in tumors of morphine-treated mice in this study complements increased metastases observed by us in subcutaneous SCK breast tumors in *A/J* mice [19]. It is likely that morphine influences tumor angiogenesis, progression, and metastases by stimulating endothelial-pericyte interaction and increased pericyte recruitment and coverage of vasculature, thus increasing resistance to antiangiogenic therapy by limiting the accessibility of drugs to the endothelium on one hand and promoting angiogenesis on the other.

While using anti-angiogenic therapy, contribution of opioids (if coadministered) to the therapeutic outcomes requires consideration. To date, there are no clinical data on the effect of morphine on cancer progression and metastases. However, OR antagonist naltrexone inhibited ovarian cancer progression in mice [38] and improved the outcome of cisplatin therapy in ovarian cancer [39]. Inhibition of advanced nonmetastatic and metastatic pancreatic cancer was also reported in patients receiving low-dose naltrexone with an antioxidant therapy with *α*-lipoic acid [40]. Morphine-induced PDGF-BB expression in HBMEC was inhibited by naltrexone [15], suggestive of an OR-mediated mechanism. Furthermore, a peripherally only acting MOR antagonist, methylnaltrexone, inhibited opioid-induced angiogenesis [20]. Therefore, coadministration of peripherally acting MOR antagonists that do not compromise morphine analgesia may improve the outcome of anti-angiogenic therapy.

In conclusion, we show that morphine stimulates PDGF-BB secretion by endothelial cells and activates PDGFR-*β* and MAPK/ERK signaling in pericytes, thus mediating endothelial-pericyte interaction. This cellular activity of morphine correlates with increased angiogenesis replete with pericyte recruitment and coverage of tumor vasculature. Thus, morphine treatment may influence the effectiveness of anti-angiogenic drugs.
